# Valproic acid induces ferroptosis and suppresses the proliferation of MDA-MB-231 cells by targeting FDFT1

**DOI:** 10.3389/fphar.2025.1540667

**Published:** 2025-05-14

**Authors:** Shuxian Zhang, Jiazhuo Liu, Yisong Yang, Ran Tao, Xin Ren, Xingzhi Zhou, Shuangping Liu

**Affiliations:** ^1^ Engineering Technology Research Center for Functional Component Utilization of Organic Natural Products, Medical College, Dalian University, Dalian, Liaoning, China; ^2^ Department of Anatomy, Medical College, Dalian University, Dalian, Liaoning, China

**Keywords:** Valproic Acid, FDFT1, breast cancer, ferroptosis, proliferation

## Abstract

**Introduction:**

Valproic acid (VPA) constitutes a branched-chain, short-chain fatty acid that serves as an antiepileptic medication. It has been increasingly recognized that VPA has presented potential anti-tumor properties, including breast cancer. However, the exploration of novel breast cancer treatment methods necessitates a more comprehensive and in-depth understanding of the novel mechanism of VPA inhibition of breast cancer. It has been proven that farnesyl-diphosphate farnesyltransferase 1 (FDFT1) participate in oncogenesis and development of cancers. However, the effect of FDFT1 on breast cancer is still obscure. Thus, it is important to investigate the potential of VPA to trigger ferroptosis in breast cancer cells via targeting FDFT1.

**Methods:**

In this study, the underlying mechanisms of VPA on ferroptosis in breast cancer cells were explored in vitro and vivo. Initially, the effects of VPA on the proliferation of breast cancer cells were assessed utilizing the Cell Counting Kit-8, cell counting, and colony formation assays. Subsequently, the ferroptosis in breast cancer cells treated with VPA were determined through the use of the Lipid Peroxidation malondialdehyde Assay Kit, reduced glutathione and oxidized glutathione disulfide Assay Kit, flow cytometry, transmission electron microscopy, and western bloting. To explore the impact of VPA in combination with ferrostatin-1, Erastin or RSL3, on MDA-MB-231 cell proliferation and ferroptosis, respective CCK-8, colony formation and WB assays were conducted. Thereafter, we assessed whether VPA facilitated ferroptosis in MDA-MB-231 cells by modulating the expression of FDFT1. Finally, the anti-breast cancer effects of VPA in vivo were validated through a xenograft mouse model, and histological examination via hematoxylin-eosin staining and immunohistochemistry staining were employed to delve into the underlying mechanisms of VPA’s inhibitory effects on breast cancer cells in vivo.

**Results and Discussion:**

The assay outcomes indicated that VPA impedes the proliferation of breast cancer cells. The findings from the ferroptosis index demonstrated that MDA-MB-231 cells are more sensitive to VPA induced ferroptosis than MCF-7 cells. Subsequent to the introduction of ferrostatin-1 (Fer-1), Erastin or RSL3, it was observed that Fer-1 reversed the ferroptosis facilitated by VPA, whereas Erastin or RSL3, in conjunction with VPA, respectively, induced ferroptosis in MDA-MB-231 cells. We revealed that the downregulation of FDFT1 enhanced proliferation and inhibited ferroptosis of MDA-MB-231 cells. Additionally, we discovered that VPA may facilitate ferroptosis in MDA-MB-231 cells by negatively modulating the levels of the solute carrier family 7 member 11 (SLC7A11) protein through the upregulation of FDFT1 expression. In conclusion, this study elucidated that VPA induced the ferroptosis of MDA-MB-231 cells via targeting FDFT1, representing a novel mechanism underlying its efficacy in potentially inhibiting breast cancer.

## 1 Introduction

According to the latest global statistics, breast cancer has surpassed lung cancer to emerge as the predominant malignancy worldwide (Sung et al., 2021). In accordance with the standards set by the North American Registry of Cancer Centers, breast cancer subtypes are delineated as follows: estrogen receptor (ER)-positive/human epidermal growth factor receptor 2 (HER2)-negative breast cancer, HR-positive/HER2-positive breast cancer, HR-negative/HER2-positive breast cancer, and HR-negative/HER2-negative breast cancer (Harbeck and Gnant, 2017). TNBC constitutes approximately 15%–20% of all breast cancer diagnoses and is distinguished by the absence of expression for ER, progesterone receptor, and HER2. This subtype of breast cancer is the most aggressive, associated with increased risk of recurrence and mortality in comparison to other breast cancer subtypes. The majority of fatalities occur during the third to fifth year post-initial treatment (Li et al., 2022; Foulkes et al., 2010). Chemotherapy serves as the predominant therapeutic modality for TNBC. However, its efficacy is often limited in the context of most metastatic cases (Denkert et al., 2017). Consequently, there is an imperative requirement for novel therapeutic approaches to surmount the therapeutic obstacles encountered in the treatment of TNBC.

Distinct from apoptosis and necrosis, ferroptosis is an iron-dependent form of cell death triggered by excessive lipid peroxidation (Cao and Dixon, 2016; Mou et al., 2019; Xu et al., 2019). The cysteine/glutamate reverse transporter, also designated as system xc-, constitutes a pivotal antioxidant mechanism within cellular compartments. It is comprised of two integral subunits: SLC7A11, and solute carrier family 3, member 2 (SLC3A2). SLC7A11 facilitates the interconversion of intracellular glutamate with extracellular cysteine in a ratio of 1:1, whereas SLC3A2 functions as a chaperone protein. The ingested cystine is processed by gamma-glutamate-cysteine ligase and glutathione synthase to synthesize reduced glutathione (GSH). This synthesized GSH is capable of reducing the levels of ROS and maintaining intracellular redox balance, thereby safeguarding cells from damage (Xie et al., 2016; Dixon et al., 2012). GSH also serves as a reducing cofactor for glutathione peroxidase 4 (GPX4), which is involved in the reduction of lipid ROS (Feng and Stockwell, 2018; Yan et al., 2021). Consequently, a deficiency of GSH or GPX4 within the cell can lead to the build-up of lipid ROS, causing membrane peroxidation damage and potentially triggering ferroptosis (Feng and Stockwell, 2018; Stockwell et al., 2017). An increasing body of research has elucidated harnessing ferroptosis to induce cancer cell death may represent a novel anti-cancer strategy. Dixon et al. discovered that the ferroptosis inducer Erastin inhibits SLC7A11 located on the cell membrane, thereby causing an excessive accumulation of lipid ROS within the cells. Subsequently, this leads to the destruction of the DNA and proteins of the cancer cells, resulting in cell death (Dixon et al., 2012). Moreover, Erastin exhibits a pronounced synergistic anti-breast cancer effect in conjunction with chemotherapy agents such as temozolomide, cisplatin, and doxorubicin (Yu et al., 2017).

FDFT1 is localized within the endoplasmic reticulum membrane and is engaged in the biological function of guiding the metabolite farnesyl pyrophosphate towards the sterol pathway for the biosynthesis of cholesterol (Tansey and Shechter, 2000). Although research has identified a correlation between accelerated cholesterol metabolism and the progression of cancer, the role of FDFT1 as a tumor suppressor or as a potential oncogene remains ambiguous (Ha and Lee, 2020). FDFT1 is significantly upregulated in ovarian cancer, prostate cancer, tongue squamous cell carcinoma, and glioma, in comparison to normal adjacent tissues (Nakae et al., 2021; Brusselmans et al., 2007; Chattopadhyay and Mallick, 2023; Facchini et al., 2018), whereas its expression is comparatively low in colon cancer, gastric cancer, and renal clear cell carcinoma (Weng et al., 2020; Zhao et al., 2021; Huang et al., 2022). In recent years, FDFT1 has been identified as a gene correlating with ferroptosis, and it is regarded as a pivotal gene for the prognostic forecast of patients afflicted with multiple malignancies (Shao et al., 2021; Yan et al., 2022; Wang et al., 2024). It has been discovered that FDFT1 serves as a molecular target mediating the induction of ferroptosis by 3β-Hydroxy-12-oleanen-27-oic Acid (ATA) in colon cancer cell line HCT116 (Tu et al., 2023). Furthermore, research indicates that FDFT1 potentially facilitates ferroptosis via the protein kinase B (AKT) signaling pathway in renal clear cell cancer cells, thereby impeding the aggressive progression of these cells (Huang et al., 2022). However, there have been scant studies in the domain of breast cancer research that have examined FDFT1.

VPA constitutes a short-chain, branched fatty acid that serves as a therapeutic agent for the management of seizures in individuals suffering from brain tumors (Barker et al., 2013). Moreover, it is efficacy in the treatment of bipolar disorder and migraines is well-documented (Lee et al., 2016). Additionally, VPA functions as a histone deacetylase inhibitor, thereby promoting the acetylation of histone and non-histone proteins. This mechanism of action not only directly curtails the proliferation of diverse tumor cells but also augments the efficacy of standard anti-tumor medications (Kiweler et al., 2020; Oi et al., 2009; Sanaei and Kavoosi, 2019). VPA has demonstrated inhibitory effects on breast cancer (Wedel et al., 2011; Li et al., 2021; Zhang et al., 2012), but additional research is required to elucidate the molecular mechanisms by which valproic acid impedes the growth of breast cancer cells. To date, no literature has described the anti-breast cancer properties of valproic acid via the pathway of ferroptosis. Consequently, the objective of this investigation was to investigate whether valproic acid inhibits the proliferation of breast cancer cells by enhancing ferroptosis, and to determine whether FDFT1 serves as a pivotal target in valproic acid’s mediation of ferroptosis in breast cancer cells.

## 2 Materials and methods

### 2.1 Cell culture

Human TNBC cell line MDA-MB-231, the normal breast epithelial cell line MCF-10A or the ER-positive breast cancer cell line MCF-7 were maintained in DMEM culture medium(Gibco) that supplied with 10% fetal bovin serum (FBS, Biological Industries) and 1% penicillin-streptomycin liquid (Solarbio). Cells were placed in a 37°C humidified atmosphere with 5% CO_2_. The corresponding reagents ferrostatin-1 (#HY-100579), Erastin (#HY-15763), and RSL3 (#HY-100218A) were purchased from MedChemExpress. VPA (#PHR1061) was purchased from Sigma.

### 2.2 Cell transfection

Small interfering RNAs (siRNA) targeting FDFT1 (siFDFT1, target sequence: GCA​GAA​TCT​TCC​CAA​CTG​T#1, GTG​CCT​GAA​TGA​ACT​TAT​A#2, GGA​GCA​GGT​ATG​TTA​AGA​A#3) and negative control oligonucleotide was obtained from RiboBio (China). The oligonucleotides were transfected to cells by using riboFECT CP Transfection (RiboBio, China) following the manufacturer’s description. An adequate number of cells were seeded into the culture wells of 6-well plates, with transfection initiated when the cell density achieved a level of 30%–50%. Subsequently, 5 µL of 20 µM siRNA stock solution (RiboBio, China) was combined with 120 µL of 1X riboFECT™CP Buffer (RiboBio, China) per well, and the mixture was carefully agitated. Thereafter, 12 µL of riboFECT™CP Reagent (RiboBio, China) was added, and the resulting solution was gently mixed before being incubated at room temperature for a duration of 15 min to form the transfection complex. Thereafter, the transfection complex was added to each well containing 1863 µL of DMEM medium. Finally, the plates were placed in a 5% CO_2_ incubator set at 37°C for cultivation.

### 2.3 Cell viability assay

Cell viability was evaluated using the CCK-8 purchased from Ape Bio (K1018). In brief, cells were planted in 96-well plates (5 × 10^3^ cells/well). After 24 h drug and transfection were added at the selected dose to the culture medium. The medium was replaced with 100 μL fresh medium containing 10 μL CCK-8 reagent and incubated in a humidified incubator (37°C, 5% CO_2_) for 2 h. The absorbance at 450 nm was checked by using a microplate reader (PerkinElmer, USA).

### 2.4 Cell counting assay

The cells were seeded into a 24-well plate at a density of 1 × 10^4^ cells per well, and three replicate wells were prepared for each treatment. These cultures were maintained in an incubator overnight. On the subsequent day, the culture medium containing 0 or 4 mmol/L concentrations of VPA was supplemented externally. Subsequently, the existing culture medium in each well was replaced, and the cultures were allowed to continue. Following a 24-h incubation period post-cell administration, the cells subjected to 24 h of drug exposure were harvested and quantified utilizing the Automated Cell Counter (BIO-RAD). thereupon, the cell counts in the wells were conducted concurrently over four consecutive days.

### 2.5 Colony formation assay

Each six-well plate was inoculated with 5 × 10^2^ cells, and three replicate wells were created within the same concentration cohort. Subsequently, the plate was situated within an incubator for the duration of the culturing process. Upon achieving adherence of the cells to the plate, a vehicle containing the therapeutic agents was introduced into the extracellular milieu, subsequently replacing the incumbent medium across all wells. The medium was exhausted approximately 14 days subsequent to the commencement of drug administration, at which point the medium within the six-well plate was discarded. Each well was delicately rinsed with 1 mL of phosphate-buffered saline (PBS) three times, followed by the addition of pre-chilled methanol, which was allowed to incubate at ambient temperature for a duration of 15 min. Post-methanol treatment, the solvent was discarded and 1 mL of 10% crystal violet (Solarbio) was added to each well, incubating for half an hour. Ultimately, the crystal violet was delicately washed with tap water, and the six-holed plate was subsequently dried on a sheet of white paper for photographic purposes.

### 2.6 Measurement of MDA

The cells were seeded in Petri dish. After treatment with different concentrations of VPA (0, 2 or 4 mM) for 24 h, the supernatant was discarded, subsequent to double-washing the Petri dishes with 1 mL of PBS each time, cellular contents from three Petri dishes were aggregated into three separately designated centrifuge tubes, subsequent to which the malondialdehyde (MDA) content within the cell lysates was determined utilizing the Lipid Peroxidation MDA Assay Kit (S0131S, Beyotime, China), in strict accordance with the provider’s recommended protocols. Finally, the OD value was measured at 532 nm by a microplate reader (Molecular Devices SPECTRAMAX, United States), and MDA levels of cells were presented as a percentage of the control.

### 2.7 Measurement of the GSH and GSSG

Each well was inoculated with 3 × 10^5^ cells and cultured overnight in a incubator. On the subsequent day, reagents were introduced to treat the cell within the pores for a duration of 24 h. Subsequently, each well was subjected to a single washing with 1 mL of PBS, and the cells were subsequently collected in a centrifuge tube. A concentration of 50 µM of C11-BODIPY dye (Invitrogen,D3861) was diluted to 5 µM in DMEM. Thereafter, the existing medium was exchanged with the diluted solution of 5 µM of C11-BODIPY dye, which was then incubated at a temperature of 37°C in the absence of light for a period of 30 min. Following three PBS washes, the sample was re-suspended in 500 µL of PBS for subsequent computer-based testing. Each set of samples was passed through a mesh screen, placed on ice, and then subjected to flow cytometric analysis utilizing a 488 nm laser at the FL1 detector via flow cytometer (Beckman).

### 2.8 Western Bloting

The cells treated with VPA were harvested and subjected to centrifugation, subsequent to which the supernatant was discarded. Protein lysate was then added to the cell pellet, followed by mixing on ice for a duration of 40 min. Upon achieving complete lysis of the cells, the lysate was subjected to low-temperature centrifugation at 12,000 rpm for 20 min in a centrifuge (Thermo Fisher), with an appropriate volume of supernatant being aspirated and the precipitate discarded. Subsequently, the BCA kit (Solarbio) was employed for protein extraction from the cell lysates, and the protein concentration in each group was determined in accordance with the manufacturer’s instructions. Afterward, calculated amounts of loading buffer and PBS were added to the extracted proteins, and the proteins were denatured by boiling in a protein sample boiler for 5 min as per the instructions. Post-cooling, the proteins were stored at −20°C in the refrigerator. sodium dodecyl sulfate polyacrylamide gel electrophoresis (SDS-PAGE) was performed with buffer solutions tailored to the varying molecular weights of the proteins. Samples were loaded onto the SDS-PAGE gel, following which the power supply’s positive and negative terminals were connected. An electrophoretic voltage of 80V was chosen and maintained until the gel run was completed. The gel was cut to the desired protein molecular weight, and the polyvinylidenefluoride (PVDF) membrane was activated in methanol for 30 s before being immersed in the membrane transfer solution. The gel was positioned in the transfer apparatus with the cathode at the bottom, followed by two layers of sponge, two layers of filter paper, the PVDF membrane, two layers of filter paper, two layers of sponge, and finally the anode. Electrophoretic transfer was conducted at a voltage of 90V for 90 min (the duration of which may be adjusted based on the molecular weight of the target protein). Upon completion of the transfer, the PVDF membrane was washed with PBST three times for 5 min each. The membrane was then incubated in a solution containing 5% skim milk (Solarbio) at room temperature for 1 hour. The anti-stock solution was serially diluted with TBST in accordance with the manufacturer’s guidelines, and subsequent incubation was performed with the respective primary antibodies at 4°C overnight. The primary antibodies employed were GAPDH (Affinity, 1:10,000), β-actin (Affinity, 1:500), TUBULIN (Affinity, 1:1000), FDFT1 (Abcam, 1:1000), GPX4 (Abcam, 1:1000), and SLC7A11 (Abcam, 1:1000; Affinity, 1:1000). Following primary antibody incubation, the membrane was subjected to a series of washes with PBST, intermittently agitated at room temperature, and cleaned three times for 15 min each. Subsequently, the secondary antibody was allowed to incubate at room temperature for 1 hour. The secondary antibody used was an Anti-rabbit IgG (Affinity, 1:5000). Upon completion of the secondary antibody incubation, the membrane was re-introduced to a shaking platform at room temperature and subjected to additional PBST washes, triply for 15 min each. Prior to visualization, the ECL chemiluminescent solution (NCM Biotech) was prepared in a 1:1 ratio of solution A to solution B, with precautions taken to prevent light exposure. The prepared luminous solution was then evenly applied to the PVDF membrane, and visualization was achieved using a gel Imager (BIO-RAD).

### 2.9 Transmission electron microscopy

The processed cells were subsequently transferred to an electron microscopy fixative (Solarbio) which was subsequently returned to room temperature. Thereafter, the cells were re-suspended, followed by fixation at 4°C for the purposes of preservation and transportation. And then wash the cells using 0.1 mL PBS for 3 times, 15 min each. Cells avoid light post fixed with 1% OsO_4_ (Ted Pella Inc 18,456) in 0.1 mL PBS for 2 h at room temperature. After remove OsO_4_, the cells are rinsed in 0.1 mL PBS for 3 times, 15 min each. Dehydrate at room temperature as followed: 30% ethanol (Sinaopharm Group Chemical Reagent Co. Ltd. 100,092) for 20 min; 50% ethanol for 20min; 70% ethanol for 20 min; 80% ethanol for 20 min; 95% ethanol for 20 min; Two changes of 100% ethanol for 20 min; Finally two changes of acetone (Sinaopharm Group Chemical Reagent Co. Ltd. 100,004) for 15 min. Resin penetration and embedding as followed: acetone:EMBed 812 (SPI 90529-77-4) = 1:1 for 2–4 h at 37°C; acetone:EMBed 812 = 1:2 overnight at 37°C; pure EMBed 812 for 5–8 h at 37°C; Pour the pure EMBed 812 into the embedding models and insert the tissues into the pure EMBed 812, and then keep in 37°C oven overnight. The embedding models with resin and samples were moved into 60°C oven to polymerize for more than 48 h. And then the resin blocks were taken out from the embedding models for standby application at room temperature. The resin blocks were sectioned into 1.5M slices using a semi-thin microtome, subsequently stained with toluidine blue, and positioned under a light microscope for positioning. The resin blocks were cut to 60–80 nm thin on the ultramicrotome (Leica, Leica UC7) and the tissues were fished out onto the 150 meshes cuprum grids with formvar film. 2% uranium acetate saturated alcohol solution avoid light staining for 8 min, rinsed in 70% ethanol for 3 times and then rinsed in ultrapure water for 3 times. 2.6% Lead citrate avoid CO_2_ staining for 8 min, and then rinsed with ultrapure water for 3 times. After dried by the filer paper, the cuprum grids were put into the grids board and dried overnight at room temperature. The cuprum grids are observed under TEM (HITACHI HT7800/HT7700) and take images.

### 2.10 Public database analysis

An analytical RNA-sequencing expression profile was retrieved from the publicly accessible database for breast cancer within the Cancer Genome Atlas (TCGA) repository via the university of Alabama at Birmingham cancer data analysis portal (UALCAN). This enabled the investigation of FDFT1 expression across normal human specimens as well as within breast cancer specimens. Subsequently, a continuous effort was made to derive additional RNA-sequencing expression profiles from the aforementioned TCGA breast cancer database within UALCAN, with the objective of examining the FDFT1 expression across diverse subtypes of breast cancer specimens.

### 2.11 Quantitative real-time PCR

Total RNA was extracted using 1 mL Triquick Reagent (Solarbio), and the RNA was reverse transcribed with a PrimeScript™ RT reagent Kit with gDNA Eraser tcDNA Synthesis Kit (Takara,RR047A). Quantitative real-time PCR was performed using TB Green^®^ Premix Ex Taq™ II Kit (Takara,RR820A). The threshold cycle (Ct) values for each gene were normalized to those of GAPDH, and the 2^−ΔΔCT^ method was used for quantitative analysis. Target genes were PCR amplified using the following primers: Q-FDFT1-F (GCA​ACG​CAG​TGT​GCA​TAT​TTT) and Q-FDFT1-R (CGC​CAG​TCT​GGT​TGG​TAA​AGG); Q-GAPDH-F (GGC​TCT​CCA​GAA​CAT​CAT​C) and Q-GAPDH-R (CTC​TTC​CTC​TTG​TGC​TCT​TG).

### 2.12 Xenograft studies

Eight SPF nude mice, aged 3–5 weeks and weighing between 16–20 g, were procured from Changsheng Bio-technology Co., Ltd. These mice were randomly allocated into two distinct groups and provided with SPF bedding and feed for a period of 1 week to ensure adaptive acclimation. To instigate subcutaneous tumor formation in the mice, a suspension of 2 × 10^6^ MDA-MB-231 cells was combined with 50 µL Matrigel (Corning) and 100 µL PBS. This cell mixture was then injected subcutaneously into the right posterior flank of the immunodeficient nude mice. Once the tumors were established, the mice were randomly assigned to two groups, each comprising five mice. One group received a daily intraperitoneal injection of valproic acid at a concentration of 150, 300 mg/kg, while the control group was administered with saline solution. Body weight and tumor dimensions were monitored daily for a duration of 10 days. Following 10 days of intraperitoneal injection, the mice were humanely euthanized. The tumors were then excised, their weights and volumes measured, and a section of each tumor was promptly fixed in 10% buffered formalin for subsequent hematoxylin-eosin (HE) staining and immunohistochemistry (IHC) analysis.

### 2.13 HE staining

The tissue sections were dehydrated sequentially in xylene, followed by anhydrous ethanol and 75% ethanol. Subsequently, they were gently washed with tap water and subjected to a high-resolution constant dye pretreatment solution for 1 min. Thereafter, the sections were stained with hematoxylin dye solution (Servicebio) for three to 5 min and gently rinsed with tap water. Hematoxylin differentiation solution (Servicebio) was applied for 2–5 s followed by tap water rinsing. This was followed by treatment with hematoxylin return to blue solution (Servicebio) for 2–5 s. After rinsing with running water, the sections were dehydrated with 95% alcohol for 1 minute and then stained with eosin dye solution (Servicebio) for 15 s. Finally, the tissue sections were immersed in anhydrous ethanol, n-butanol, and xylene successively, and sealed with a neutral gum. Microscopic imaging and analysis were subsequently conducted.

### 2.14 IHC staining

Put the sections into environmentally friendly dewaxing solution I 10 min → Environmentally friendly dewaxing solution II 10 min → Environmentally friendly dewaxing solution III 10 min → anhydrous ethanol I 5 min → anhydrous ethanol II 5 min → anhydrous ethanol III 5 min → distilled water in turn. During this process, the buffer should be prevented from excessive evaporation and should not be dried. After natural cooling, the slide was placed in PBS and washed by shaking on the decolorizing shaker for 3 times, 5 min each time. The slices were placed in 3% hydrogen peroxide solution, incubated at room temperature away from light for 25 min, and the slides were placed in PBS and washed three times on a decolorizing shaking table for 5 min each time. The tissue was uniformly covered with 3% BSA in the tissue chemical circle and closed at room temperature for 30 min. Gently shake off the sealing solution, add PBS to the section in a certain proportion of primary antibody, and the section is placed flat in a wet box at 4°C for overnight incubation. The slide was placed in PBS and washed by shaking on the decolorizing shaker for 3 times, 5 min each time. After the slices were slightly dried, the tissue was covered with the secondary antibody (HRP label) of the corresponding species of the primary antibody, and incubated at room temperature for 50 min. The slide was placed in PBS and washed by shaking on the decolorizing table for 3 times, 5 min each time. After the sections were slightly dried, the freshly prepared DAB color developing solution was added into the circle. The color developing time was controlled under the microscope. The positive color was brown and yellow, and the sections were rinsed with tap water to terminate the color development. Hematoxylin re-staining for about 3 min, washing with tap water, hematoxylin differentiation solution for a few seconds, rinse with tap water, hematoxylin return to blue solution, and rinse with running water. Put the slices into 75% alcohol for 5 min → 85% alcohol for 5 min → anhydrous ethanol for 5 min → anhydrous ethanol for 5 min → n-butanol for 5 min → xylene for 5 min to dehydrate and transparent, take the slices out of xylene to dry slightly, and seal the slices with glue. The results are interpreted under a white light microscope.

### 2.15 Statistical analysis

The experimental data were analyzed employing Graphpad Prism version 6.01 software. The mean value of the recorded data is represented as the mean ± standard deviation. The independent two-sample t-test was conducted for the comparison between the two sets of data, while one-way analysis of variance (ANOVA) was utilized for the comparison among three or more groups of data. The p-value of less than 0.05 was considered indicative of statistically significant differences.

## 3 Results

### 3.1 VPA suppresses proliferation of breast cancer cells *in vitro*

**FIGURE 1:**
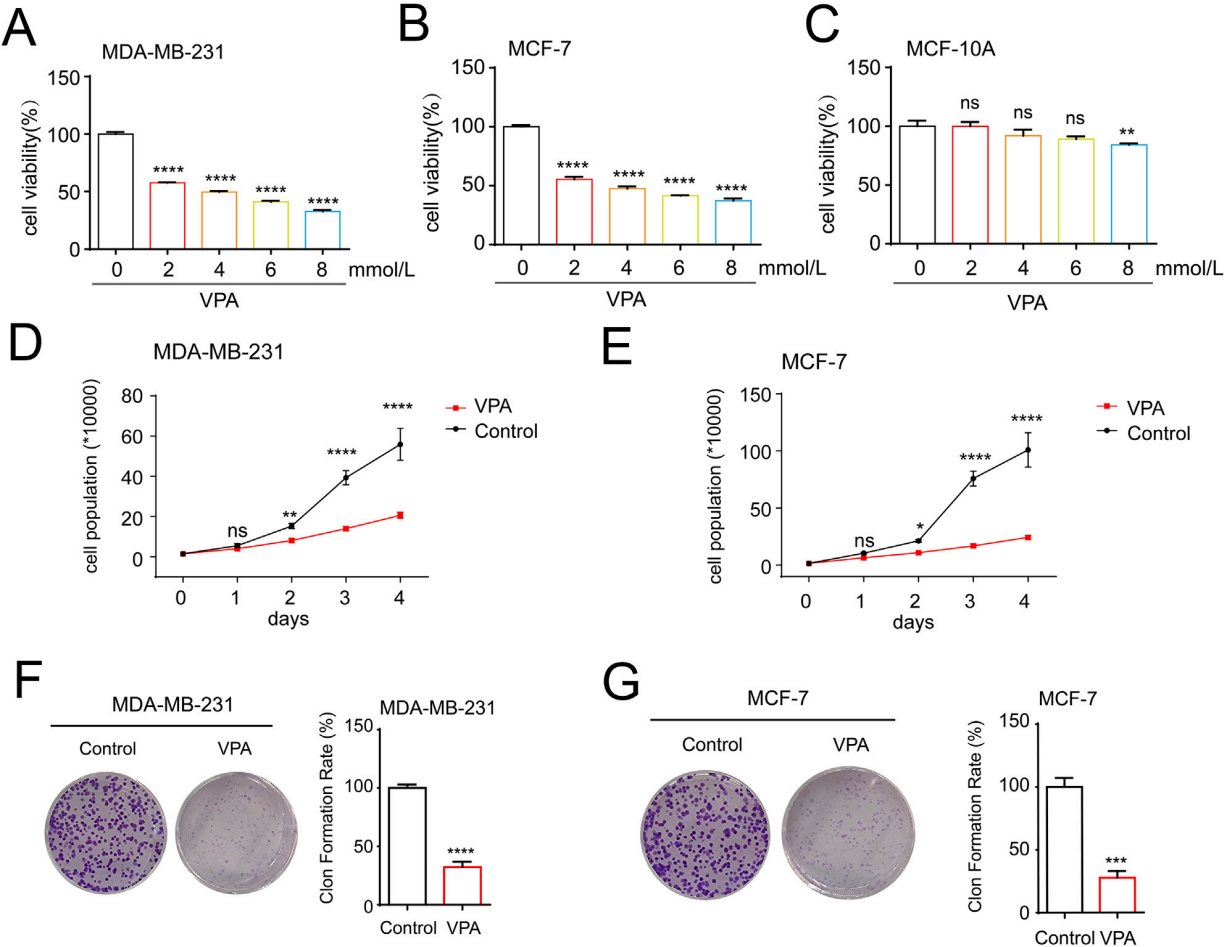
VPA suppresses proliferation of breast cancer cells *in vitro*. **(A)** MDA-MB-231 cells were treated with VPA (0, 2, 4, 6, 8 mM) for 24 h, and cell viability was assayed using CCK-8 assay. **(B)** MCF-7 cells were treated with VPA (0, 2, 4, 6, 8 mM) for 24 h, and cell viability was assayed using CCK-8 assay. **(C)** MCF-10A cells were treated with VPA (0, 2, 4, 6, 8 mM) for 24 h, and cell viability was assayed using CCK-8 assay. **(D)** MDA-MB-231 cells were treated with VPA (4 mM) for 0, 1, 2, 3 or 4 days, and the number of cells were counted. **(E)** MCF-7 cells were treated with VPA (4 mM) for 0, 1, 2, 3 or 4 days, and the number of cells were counted. **(F)** MDA-MB-231 cells were treated with VPA (2 mM) for 14 days, and count the number of cell clones after staining with crystal violet. **(G)** MCF-7 cells were treated with VPA (2 mM) for 14 days, and count the number of cell clones after staining with crystal violet. Data are presented as means ± sd (ns. not significant. *p < 0.05, **p < 0.01, ***p < 0.001 and ****p < 0.0001).

To detect the cytotoxicity of VPA toward TNBC cell line MDA-MB-231 and luminal A cell line MCF-7 *in vitro*, we treated breast cancer cells with various concentrations of VPA (from 2 to 8 mM) for 24 h and determined the cell viability using CCK-8 assays. VPA exerted dose-dependent toxicity and proliferation inhibitory effects on MDA-MB-231 and MCF-7 cells (Figures 1A, B) but not normal breast epithelial cell line MCF-10A (Figure 1C), indicating VPA possessed selective anti-breast cancer properties. We used cell counting analysis to find that VPA treatment inhibited 63.04% (MDA-MB-231) and 71.93% (MCF-7) cell numbers in Figures 1D, E. Colony formation analysis showed that VPA treatment significantly inhibited the proliferation of MDA-MB-231 (67.85%) and MCF-7 (72.26%) cells (Figures 1F, G). Therefore, these data suggest that VPA shows potent anti-breast cancer activity, suggesting its potential as a therapeutic drug.

### 3.2 The TNBC cell line MDA-MB-231are more sensitive to ferroptosis than the luminal A cell line MCF-7, and VPA is identified as a potent ferroptosis inducer

**FIGURE 2:**
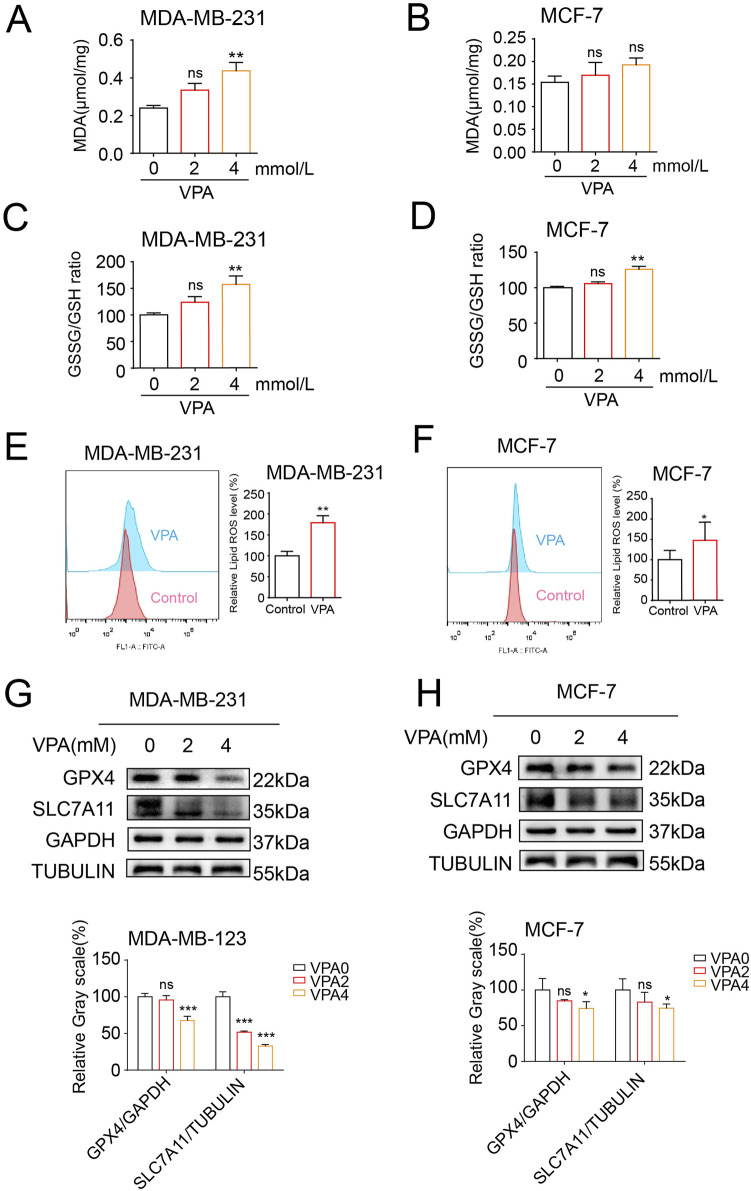
The TNBC cell line MDA-MB-231are more sensitive to ferroptosis than the luminal A cell line MCF-7, and VPA is identified as a potent ferroptosis inducer. **(A)** MDA-MB-231 cells were treated with VPA at 0 or 4 mM for 24 h, and the levels of MDA were assayed. **(B)** MCF-7 cells were treated with VPA at 0 or 4 mM for 24 h, and the levels of MDA were assayed. **(C)** MDA-MB-231 cells were treated with VPA at 0 or 4 mM for 24 h, and the GSH and GSSG contents were assayed. **(D)** MCF-7 cells were treated with VPA at 0 or 4 mM for 24 h, and the GSH and GSSG contents were assayed. **(E)** MDA-MB-231 cells were incubated with VPA (0 or 4 mM) for 24 h, stained with C11-BODIPY, and then subjected to flow cytometry analysis. **(F)** MCF-7 cells were incubated with VPA (0 or 4 mM) for 24 h, stained with C11-BODIPY, and then subjected to flow cytometry analysis. **(G)** MDA-MB-231 cells were treated with VPA at 0 or 4 mM for 24 h SLC7A11 and GPX4 protein expression was measured via WB. **(H)** MCF-7 cells were treated with VPA at 0 or 4 mM for 24 h SLC7A11 and GPX4 protein expression was measured via WB. Data are presented as means ± sd (ns. not significant. *p < 0.05, **p < 0.01, and ***p < 0.001).

To determine whether VPA exerted an anti-cancer effect by promoting ferroptosis in breast cancer cells, we first investigated the effects of VPA on the lipid peroxidation of ferroptosis. We measured the levels of MDA and oxidized glutathione disulfide (GSSG)/GSH ratio in MDA-MB-231 and MCF-7 cells upon VPA treatment given that the levels of MDA and GSSG/GSH ratio increased when lipid peroxidation occurs. As shown in Figures 2A, B, with increasing VPA concentration, MDA content increased by 19.7 and 4.1 μmol/mg in MDA-MB-231 and MCF-7 cells, respectively. And intracellular GSSG/GSH ratio increased by 57.34% (MDA-MB-231) and 26.01% (MCF-7) in Figures 2C, D. Additionally, we employed BODIPY 581/591 undecanoic acid to detect lipid ROS upon VPA treatment. As shown by flow cytometry, a more significant change in C11-BODIPY signaling was observed in MDA-MD-231 cells following VPA treatment compared to MCF-7 cells, indicating lipid ROS induction (Figures 2E, F). To elucidate the underlying mechanisms by which VPA promotes ferroptosis, we examined the expression levels of key ferroptosis pathway components, SLC7A11 and GPX4. Our findings indicated that VPA markedly diminished the protein levels of SLC7A11 and GPX4 in MDA-MB-231 cells, as compared to the MCF-7 cells. (Figures 2G, H). Collectively, these data demonstrate that VPA potently facilitates ferroptosis in MDA-MB-231 cells relative to MCF-7 cells, and that the induction of ferroptosis in breast cancer cells by VPA is mediated through the SLC7A11-GPX4 signaling pathway.

### 3.3 VPA inhibits the proliferation and viability of MDA-MB-231 cells through the ferroptosis pathway

**FIGURE 3:**
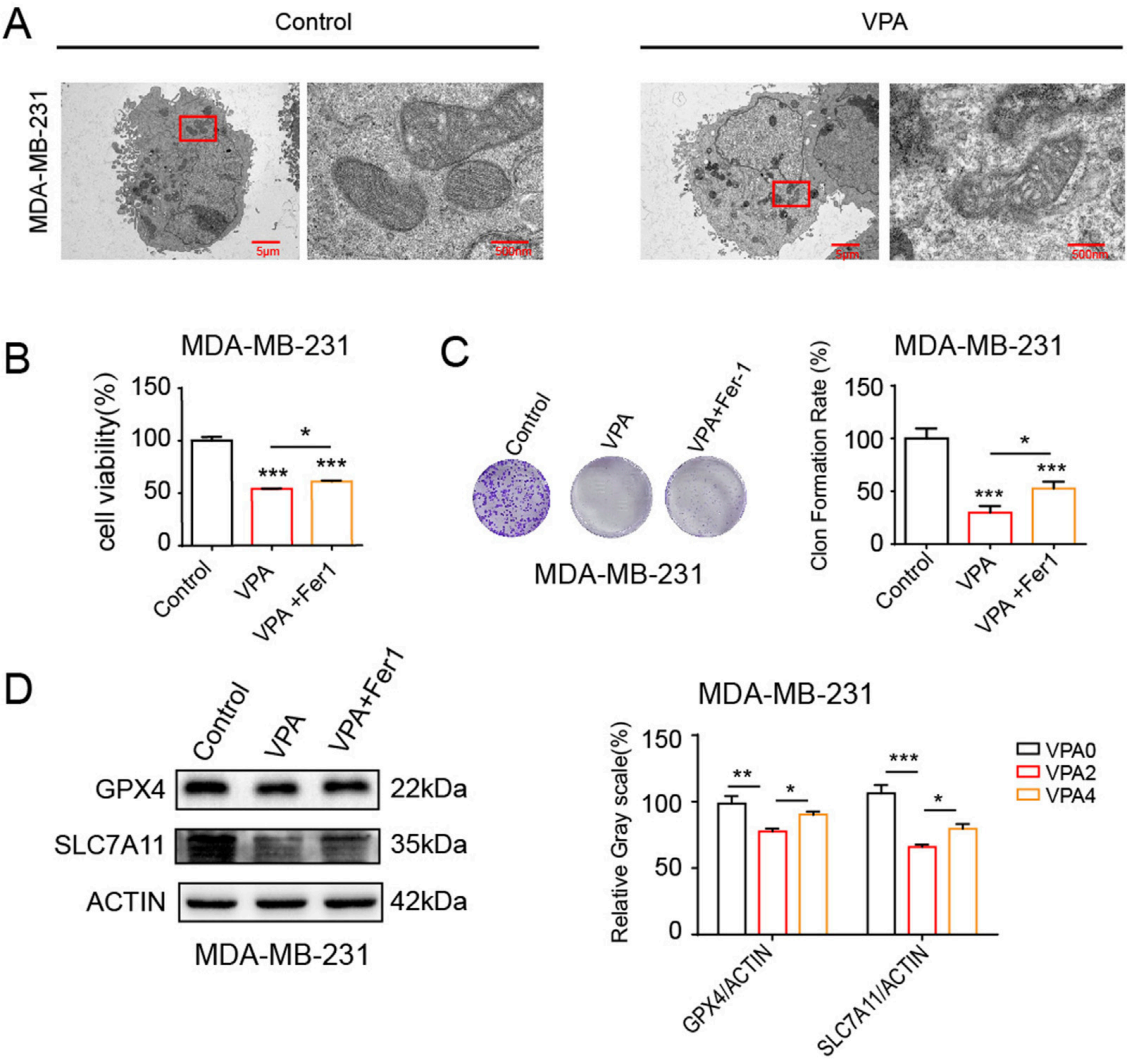
VPA inhibits the proliferation and viability of MDA-MB-231 cells through the ferroptosis pathway. **(A)** MDA-MB-231 cells morphology was observed via TEM after cells were treated with VPA (0 or 4 mM) for 24 h **(B)** MDA-MB-231 cells were cultured in 96-well plates. Fer-1 (500 nM) was incubated with VPA (4 mM) for 24 h. The viability of MDA-MB-231 cells was tested by CCK-8 assay. **(C)** MDA-MB-231 cells were cultured in 6-well plates. Fer-1 (500 nM) was incubated with VPA (2 mM) for 24 h. The proliferation of MDA-MB-231 cells was tested by colony formation assay. **(D)** MDA-MB-231 cells treated with VPA (4 mM) were incubated with Fer-1 (500 nM) for 24 h. Then the expressions of GPX4, SLC7A11 were detected using WB. Data are presented as means ± sd (*p < 0.05, **p < 0.01 and ***p < 0.001).

Based on the aforementioned discoveries, our research persists in examining MDA-MB-231 cells, which exhibit heightened sensitivity to VPA-induced ferroptosis. At first, we investigated the effects of VPA on the morphological characteristics of ferroptosis in MDA-MB-231 cells. One of the unique morphological characteristics of ferroptosis is a decrease in mitochondrial volume and an increase in membrane density (Stockwell et al., 2020). TEM showed that VPA induced a decrease in mitochondrial volume and an increase in membrane density in MDA-MB-231 cells (Figure 3A). To investigate the role of VPA-induced ferroptosis, MDA-MB-231 cells were pre-treated with Fer-1, a known inhibitor of ferroptosis. revealed a modest increase in cell viability (approximately 13.3% and 22.74%, respectively) as illustrated in Figures 3B, C. The above results prove that the anti-cancer activity of VPA via ferroptosis induction. Furthermore, when combined with Fer-1, VPA-induced the inhibition of the levels of SLC7A11 and GPX4 was abolished (Figure 3D). Collectively, these data demonstrate VPA affects the proliferation and viability of MDA-MB-231 cells through the ferroptosis pathway.

### 3.4 VPA synergistically enhances the anti-cancer activity and ferroptosis of ferroptosis inducers

**FIGURE 4:**
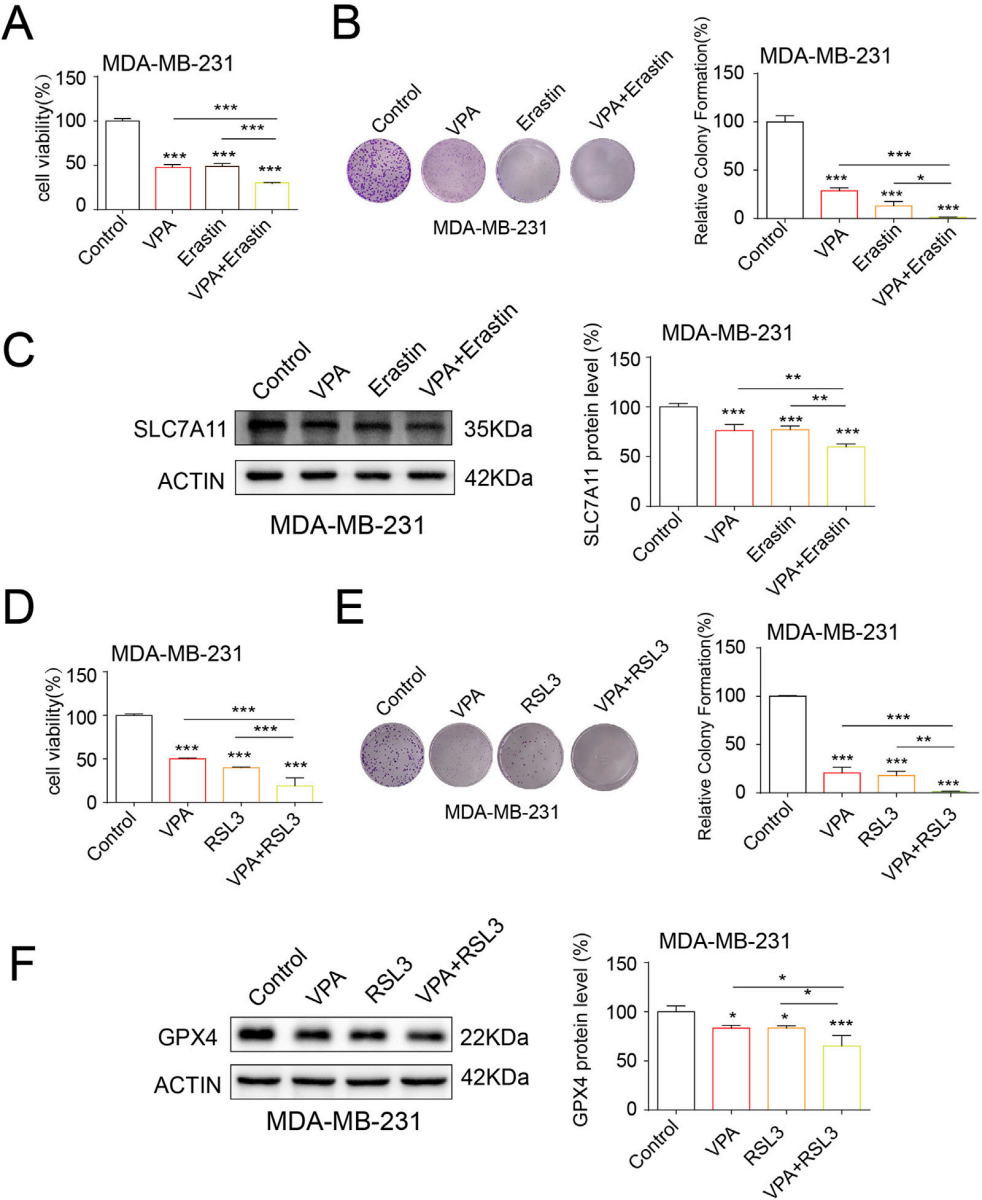
VPA synergistically enhances the anti-cancer activity and ferroptosis of ferroptosis inducers. **(A)** MDA-MB-231 cells treated with VPA (0 mM), VPA (4 mM), Erastin (5 μM), or VPA (4 mM) with Erastin (5 μM) for 24 h and then subjected to CCK-8 assay. **(B)** MDA-MB-231 cells treated with VPA (0 mM), VPA (2 mM), Erastin (5 μM), or VPA (2 mM) with Erastin (5 μM) for 24 h and then subjected to colony formation assay. **(C)** MDA-MB-231 cells treated with VPA (0 mM), VPA (4 mM), Erastin (5 μM), or VPA (4 mM) with Erastin (5 μM) for 24 h. Then the expression of SLC7A11 were detected using WB. **(D)** MDA-MB-231 cells treated with VPA (0 mM), VPA (4 mM), RSL3 (100 nM), or VPA (4 mM) with RSL3 (100 nM) for 24 h and then subjected to CCK-8 assay. **(E)** MDA-MB-231 cells treated with VPA (0 mM), VPA (2 mM), RSL3 (100 nM), or VPA (2 mM) with RSL3 (100 nM) for 24 h and then subjected to colony formation assay. **(F)** MDA-MB-231 cells treated with VPA (0 mM), VPA (4 mM), RSL3 (100 nM), or VPA (4 mM) with RSL3 (100 nM) for 24 h. Then the expression of GPX4 were detected using WB. Data are presented as means ± sd (*p < 0.05, **p < 0.01 and ***p < 0.001).

Ferroptosis inducers such as Erastin and RSL3 have been demonstrated to curtail SLC7A11 and GPX4 expressions, respectively, thereby facilitating ferroptosis (Dixon et al., 2012). To verify VPA treatment-induced ferroptosis, we asked whether VPA would increase MDA-MB-231 cells sensitivity to ferroptosis inducers, Erastin and RSL3. We performed cell viability assay and colony formation assay to assess the effect of treatment. In Figure 4A, the cell viability assay showed that when MDA-MB-231 cells treated with VPA were further exposed to Erastin, the cell proliferation rate was significantly higher than that of the group of cells treated with VPA only. Similarly, Figure 4B shows that cell viability was significantly increased after the addition of RSL3 to VPA-treated MDA-MB-231 cells, indicating enhanced cell proliferation. In the cell colony formation assay, Figure 4C shows that in MDA-MB-231 cells, the combination of VPA and Erastin resulted in a significant increase in the number and size of cell colonies, indicating a significant enhancement of cell proliferation. Correspondingly, Figure 4D shows that treatment of VPA-pretreated MDA-MB-231 cells with RSL3 also resulted in a significant increase in cell colony formation. Likewise, Erastin and RSL3 treatment synergized with VPA and significantly suppressed SLC7A11 and GPX4 expression, respectively (Figures 4E, F). Collectively, these data demonstrate the anti-TNBC activity of VPA via ferroptosis induction., and combine treatment with VPA and ferroptosis inducers result in synergistic response in MDA-MB-231 cells.

### 3.5 The knockdown of FDFT1 stimulates cell proliferation and inhibits ferroptosis in MDA-MB-231 cells

**FIGURE 5:**
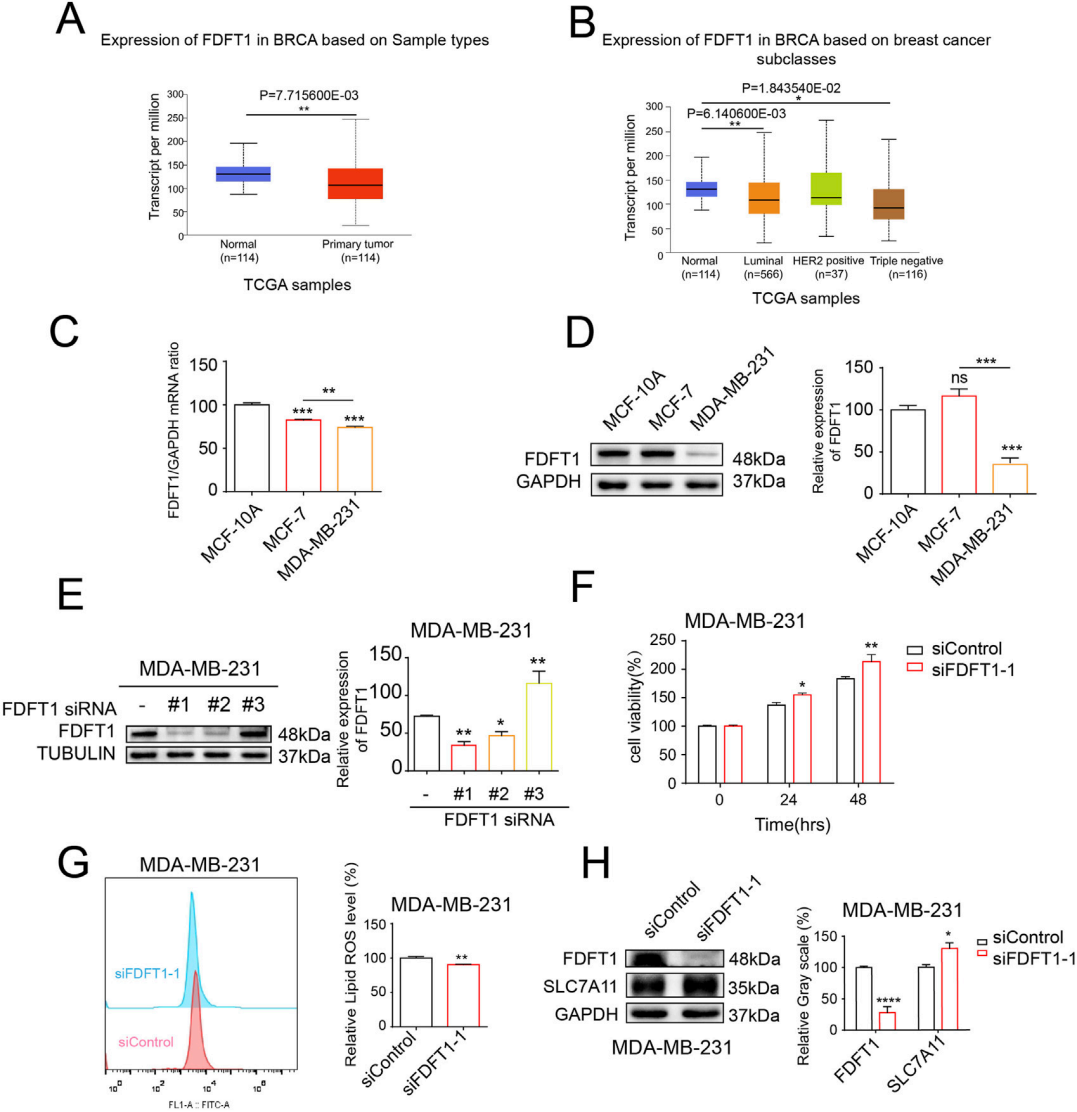
The knockdown of FDFT1 stimulates cell proliferation and inhibits ferroptosis in MDA-MB-231 cells. **(A)** FDFT1 expression statuses in breast cancer compared to normal samples were obtained from the TCGA database. **(B)** FDFT1 expression statuses in TNBC compared to non-TNBC samples were obtained from the TCGA database. **(C)** The mRNA expression levels of FDFT1 were quantified via qRT-PCR in the normal breast epithelial cell line MCF-10A, the ER-positive breast cancer cell line MCF-7, and the TNBC cell line MDA-MB-231. **(D)** The protein expression levels of FDFT1 were quantified via WB in the normal breast epithelial cell line MCF-10A, the luminal A breast cancer cell line MCF-7, and the basal-like breast cancer cell line MDA-MB-231. **(E)** The impact of FDFT1 siRNA on FDFT1 protein expression levels in TNBC cells, as compared to negative controls. **(F)** MDA-MB-231 cells were transfected with siFDFT1-1 or siControl for 0, 24, 48 h, and cell viability was assayed. **(G)** MDA-MB-231 cells were transfected with siFDFT1-1 or siControl for 24 h, stained with C11-BODIPY, and then subjected to flow cytometry analysis. **(H)** MDA-MB-231 cells were transfected with siFDFT1-1 or siControl for 24 h. Then the expression of SLC7A11 were detected using WB. Data are presented as means ± sd (*p < 0.05, **p < 0.01, ***p < 0.001 and ****p < 0.0001).

In order to acquire an initial insight into FDFT1 expression patterns in human breast cancer, we screened TCGA databases via UALCAN and discovered that the expression of FDFT1 is reduced in breast cancer when constrasted with that observed in normal breast cancer. Additionally, compared to non-TNBC, FDFT1 expression was potently downregulated in TNBC (Figures 5A, B). Furthermore, the levels of mRNA and protein for FDFT1 in the normal breast epithelial cell line MCF-10A, the ER-positive breast cancer cell line MCF-7, and the TNBC cell line MDA-MB-231 were determined via WB and qRT-PCR assays. The findings revealed that the expression levels of FDFT1 mRNA and protein were significantly decreased in MDA-MB-231 cells, whereas they were comparatively elevated in MCF-10A and MCF-7 cells (Figures 5C, D). To delve deeper into the functional significance of FDFT1 in modulating proliferation and ferroptosis in the TNBC cell line MDA-MB-231, we employed three distinct siRNAs, designated as 1, 2, and 3, to ablate FDFT1 expression in MDA-MB-231 cells. The experimental findings revealed that FDFT1 siRNA sequence No. 1 exerted the most pronounced impact on the suppression of FDFT1 protein expression compared to the negative control.in MDA-MB-231 cells (Figure 5E). As expected, the cell viabilities of MDA-MB-231 cells were induced by the treatment of the knockdown of FDFT1 (Figure 5F). Therefore, these findings indicate that FDFT1 may function as a tumor suppressor in TNBC cell line MDA-MB-231. Additionally, the levels of lipid ROS were decreased by the knockdown of FDFT1 in MDA-MB-231 cells (Figure 5G). The WB assay confirmed that the knockdown of FDFT1 markedly upregulated SLC7A11 levels in MDA-MB-231 cells (Figure 5H), this indicated that the knockdown of FDFT1 confers MDA-MB-231 cells resistant to ferroptosis, through the upregulation of SLC7A11 expression. Together these results indicate that FDFT1 represses proliferation and induces ferroptosis of MDA-MB-231 cells *in vitro*.

### 3.6 VPA induces ferroptosis by upregulating FDFT1 in MDA-MB-231 cells

**FIGURE 6:**
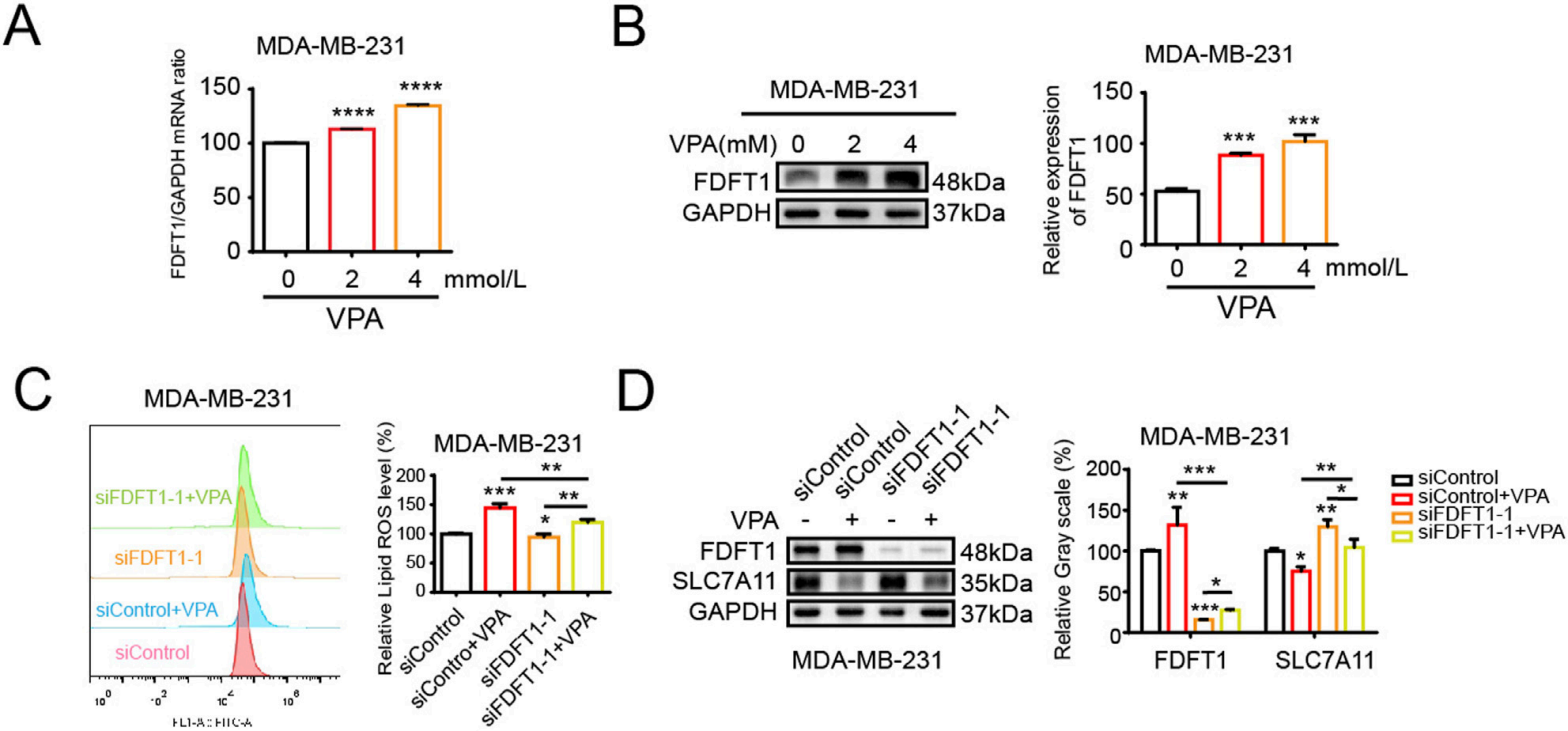
VPA induces ferroptosis by upregulating FDFT1 in MDA-MB-231 cells. **(A)** MDA-MB-231 cells were treated with VPA at 0 or 4 mM for 24 h. FDFT1 mRNA expression was measured via qRT-PCR. **(B)** MDA-MB-231 cells were treated with VPA at 0 or 4 mM for 24 h. FDFT1 protein expression was measured via WB. **(C)** MDA-MB-231 cells transfected with siFDFT1-1 or siControl were incubated with VPA (4 mM) for 24 h, stained with C11-BODIPY, and then subjected to flow cytometry analysis. **(D)** MDA-MB-231 cells lines transfected with siFDFT1-1 or siControl were incubated with VPA (4 mM) for 24 h. The protein levels of FDFT1 and SLC7A11 were measured via WB. Data are presented as means ± sd (*p < 0.05, **p < 0.01, ***p < 0.001 and ****p < 0.0001).

Our above findings suggest that VPA could induce TNBC cell ferroptosis and FDFT1 confers cells facilitative to ferroptosis. Consequently, it is speculated that a potential correlation may exist between VPA and FDFT1, our findings from qRT-PCR and WB assays indicated that VPA augmented the mRNA and protein levels of FDFT1 in MDA-MB-231 cells (Figures 6A, B). Next, we showed that the treatment of VPA stimulated lipid ROS levels and inhibited SLC7A11 levels of MDA-MB-231 cells while siRNA-mediated knockdown of FDFT1 was able to reverse the effect of VPA on MDA-MB-231 ferroptosis *in vitro* (Figures 6C, D). Taken together, these data suggest that inhibition of FDFT1 blocks VPA-induced ferroptosis of MDA-MB-231 cells, and VPA may facilitate ferroptosis in MDA-MB-231 cells by negatively modulating the expression of SLC7A11 via the upregulation of FDFT1 levels.

### 3.7 VPA attenuates proliferation of MDA-MB-231 cells *in vivo*

**FIGURE 7:**
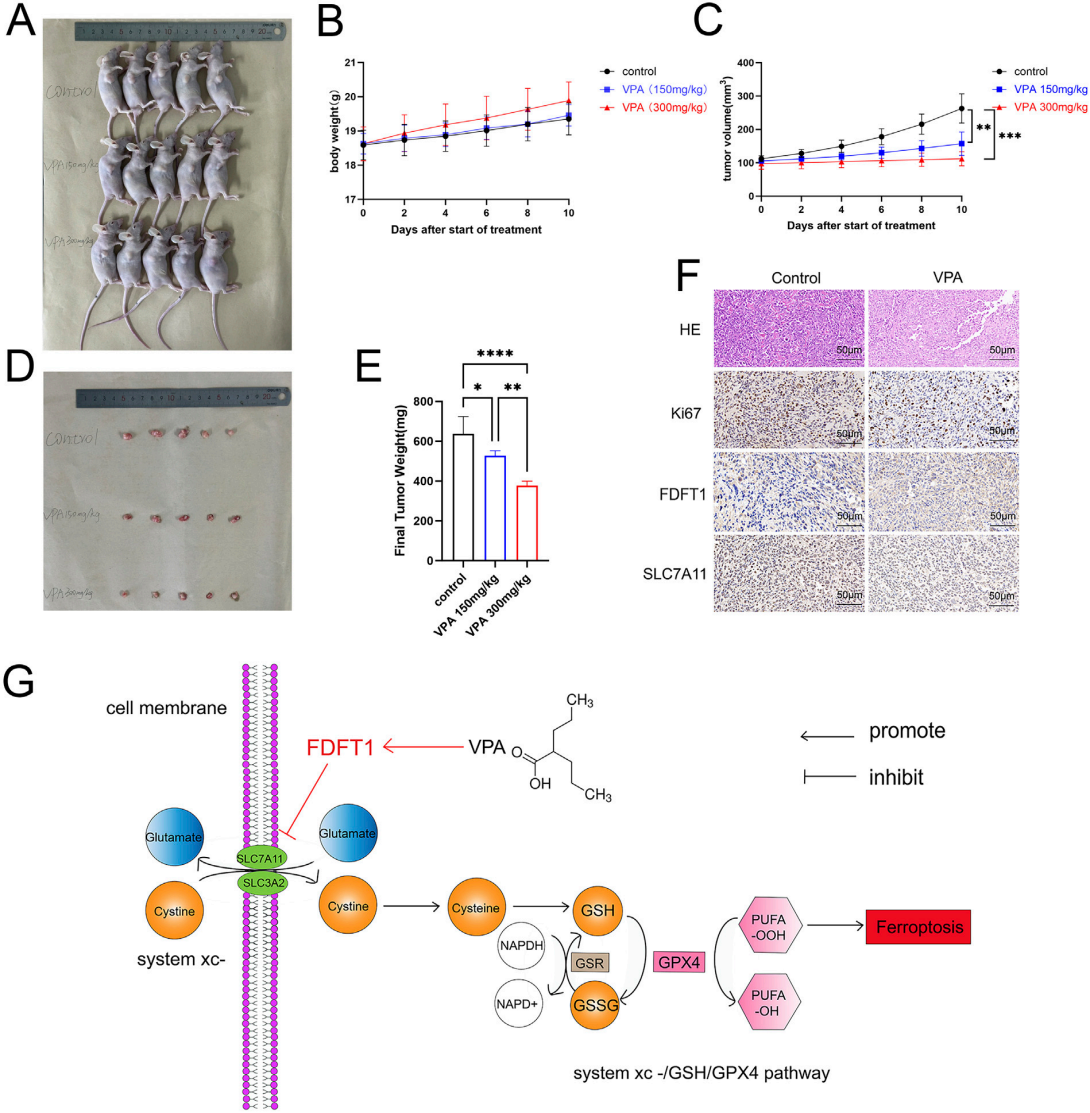
VPA attenuates proliferation of MDA-MB-231 cells *in vivo*. **(A)** Comparison of tumor size. **(B)** Body weight curves. **(C)** Tumor growth curves. **(D)** Isolated subcutaneous tumors. **(E)** Final Tumor weight. **(F)** HE, Ki67, FDFT1, and SLC7A11-stained images of tumor tissues. **(G)** VPA elicits ferroptosis in TNBC cell line MDA-MB-231 by targeting the FDFT1-SLC7A11 axis. Data are presented as means ± sd (***p < 0.001 and ****p < 0.0001).

Our study examined whether VPA impedes the proliferation of the TNBC cell line MDA-MB-231 *in vivo*. MDA-MB-231 cells were implanted subcutaneously into the right flank of immunodeficient nude mice. One week later, tumor bearing mice were randomly divided into two groups and subsequently treated with physiological saline or VPA for 10 days. Throughout this interval, the body mass of nude mice and the histological features of the tumors were documented prior to each intraperitoneal injection. The results showed that there were significant differences in tumor characteristics compared to the control group. As shown in Figure 7A, visually, the tumor volume of the nude mice treated with VPA looked reduced, suggesting a potential inhibitory effect of VPA on tumor growth. Figure 7B plots the change in body weight of mice over time, further emphasizing the sustained weight loss of mice in the VPA-treated group compared to the control group. Meanwhile, Figure 7C shows that the tumor volume of mice in the VPA-treated group was subsequently reduced. Moreover, the body weight and tumor volume of mice showed a dose-dependent change with the concentration of VPA used (Figures 7A–C). Eventually, the tumor was excised and subsequent imaging, dimensional assessments, and weight determinations were conducted, unsurprisingly, VPA significantly suppressed tumor growth compared with the control group (Figures 7D, E). Furthermore, HE staining was conducted on mouse tumor sections, which revealed that the tumor cells in the control group were intact and densely packed with intense chromatin staining. Following VPA treatment, the tumor cells demonstrated a more disorganized arrangement, exhibited pale chromatin staining, and the majority of the tissues presented evident signs of degeneration and necrosis. Additionally, through IHC staining, we detected the expression of SLC7A11, FDFT1, and Ki67, a molecular marker of cellular proliferation. Our data demonstrated that mice treated with VPA exhibited reduced Ki67 expression in comparison to the control group. Moreover, in alignment with *in vitro* findings, mice subjected to VPA treatment exhibited reduced expression of SLC7A11 and elevated expression of FDFT1 (Figure 7F). The data indicate that VPA may facilitate ferroptosis in MDA-MB-231 cells by repressing the expression of SLC7A11, thereby contributing to the inhibition of tumor growth *in vivo*.

## 4 Discussion

The TNBC are prevalent female malignancies with increasing occurrence incidence and metastasis, significantly affecting the health and life quality of women globally (Foulkes et al., 2010; Denkert et al., 2017). Ferroptosis is a type of cell death that is contingent upon iron availability, brought about by the build-up of lipid peroxides (Dixon et al., 2012). Studies have indicated that the propensity of TNBC cells to undergo ferroptosis has a significant influence on the progression, prognosis, and development of drug resistance in these tumors (Feng and Stockwell, 2018). Consequently, therapeutic agents that specifically target the regulatory mechanisms of ferroptosis may potentially modulate this process in TNBC cells, thereby enhancing the efficacy of treatment regimens for this disease. VPA represents a widely implemented antiepileptic pharmaceutical within clinical settings, and its capacity to inhibit histone deacetylation renders it a pivotal candidate for the advancement of novel anticancer therapeutics (Kiweler et al., 2020; Oi et al., 2009; Sanaei and Kavoosi, 2019). Previous research has demonstrated that valproic acid can modulate the compromised mitochondrial function by suppressing the expression of mitochondrial elongation factor 1 (MIEF1), thus inhibiting the proliferation of breast cancer cells (Du et al., 2022). Additionally, cellular ferroptosis is typified by a decrease in mitochondrial cristae or the presence of cavitation, accompanied by an increase in mitochondrial membrane density (Stockwell et al., 2020). Consequently, it is hypothesized that the inhibitory effect of valproic acid on the proliferation of TNBC cells may be implicated in the regulatory mechanism of ferroptosis by VPA.

In the initial phase of our investigation, we conducted an *in vitro* study to examine the impact of VPA on the viability of breast cancer cells. For this examination, we utilized the TNBC cell line MDA-MB-231 and luminal A cell line MCF-7. The findings from the CCK-8 assay revealed that valproic acid exerted a dose-dependent inhibitory effect on the activity of these cells. Subsequent experiments involving cell counting and clonal formation assessments further corroborated these results, demonstrating a significant reduction in the proliferation capacity of the MDA-MB-231 and MCF-7 cells treated with VPA, as compared to the control group. Additionally, we have identified the ferroptosis index and observed that after VPA treatment, the increase in MDA levels in MDA-MB-231 cells was more significant compared to MCF-7 cells. Consistent with this finding, flow cytometry analysis also showed a more significant increase in lipid ROS levels in MDA-MB-231 cells treated with VPA. Furthermore, electron microscopic observations revealed a distinctive feature of mitochondrial alterations, including a reduction or even a complete disappearance of mitochondrial cristae and an increase in mitochondrial membrane density in the MDA-MB-231 cells exposed to VPA. These observations provide evidence for the occurrence of ferroptosis in MDA-MB-231 cells following valproic acid administration. Finally, WB analysis demonstrated that VPA significantly reduced the protein expressions of GPX4 and SLC7A11, which are negative regulators of ferroptosis in cells. This finding further implies that VPA may potentially promote ferroptosis in MDA-MB-231 cells. To delve deeper into the mechanism of ferroptosis induced by VPA in breast cancer cells, we conducted experiments where MDA-MB-231 cells were exposed to a ferroptosis inhibitor, Fer-1, in conjunction with VPA. The findings revealed that Fer-1 effectively reversed the ferroptosis triggered by VPA, suggesting that valproic acid impacts the proliferation of MDA-MB-231 cells via the ferroptosis pathway. When breast cancer cells were exposed to a combination of Erastin, RSL3, and VPA, a synergistic enhancement of ferroptosis was observed, concomitantly reducing the cells' proliferation. These outcomes indicate that valproic acid can potently impede the proliferation of MDA-MB-231 cells and foster ferroptosis by modulating the SLC7A11/GPX4 pathway.

FDFT1 constitutes a pivotal molecule that dictates the direction of sterol biosynthesis (Tansey and Shechter, 2000). In recent years, FDFT1 has been pinpointed as a gene intertwined with ferroptosis, and it is acclaimed as a critical factor for forecasting the clinical outcomes of patients grappling with diverse malignancies (Huang et al., 2022; Shao et al., 2021; Yan et al., 2022). Nevertheless, the underlying mechanism of FDFT1’s involvement in the progression of breast cancer and the facets of ferroptosis remains elusive. Our investigation revealed that the expression levels of FDFT1 in breast cancer specimens were notably reduced when compared to those in normal tissues, with the lowest expression observed in triple-negative breast cancer. These findings align with the trends observed in the UALCAN, indicating a potential role for FDFT1 as a tumor suppressor in TNBC. Recent studies have identified FDFT1 as a potential driver of ferroptosis induction in various tumors (Duan et al., 2025; Xia et al., 2022). Notably, FDFT1 was a positive regulator of iron prolapse in Asian HCC patients, whereas it was a negative regulator in white HCC patients (Xia et al., 2022). The expression levels of FDFT1 in CRC cell lines (HCT8, HCT116, SW480, SW620, SW1463, RKO and HT29) were significantly lower than those of normal cell lines NCM460 and FHC. Similarly, our data likewise found that the expression of FDFT1 in tumor cell lines was not entirely consistent. Interestingly, the effect of FDFT1 on cancer is heterogeneous, serving opposite roles in different tumor cells (Yang et al., 2014; Cai et al., 2025; Brusselmans et al., 2007; Tuzmen et al., 2025). 3β-Hydroxy-12-oleanolic acid-27-oleanolic acid (ATA) is an antitumor agent isolated from the rhizome of fallen bamboo. Existing studies have shown that FDFT1 is a key upstream target that regulates the proliferation of ATA anti-HCT116 cells, in which FDFT1 plays an oncogene effect (Tu et al., 2023). This is different from in our results, which shows that FDFT1 has two sides in cancer cells. And when it is used as a therapeutic target, the effects of different drugs on it may be completely opposite. Therefore, there may be limitations in developing FDFT1 as a cancer therapeutic target, mainly attributed to its heterogeneity in tumors. Meanwhile, our results showed that valproic acid significantly inhibited the growth of MDA-MB-231 cells in these mice *in vivo*, and the expression of Ki67 and SLC7A11 in the tumor tissues was significantly downregulated after the administration of valproic acid, which was consistent with our *in vitro* experimental results. This suggested that VPA may regulate the expression of SLC7A11 through FDFT1, which triggered the ferroptosis of MDA-MB-231 cells and exerted an inhibitory effect on their proliferation *in vitro* and *in vivo* (Figure 7G). Similar to our results, reduced SLC7A11 levels sensitize cancer cells to ferroptosis in different tumors. The downregulation of Fatty acid synthase promotes ferroptosis, mainly by decreasing SLC7A11 in cervical cancer, and targeting FASN enhances cisplatin sensitivity in cervical cancer (Wang et al., 2025). In addition, MSL1, a scaffolding protein of the histone acetyltransferase complex, promoted erastin-induced ferroptosis in HCT116 and SW480 cells via the KCTD12-SLC7A11 axis (Luo et al., 2025). Interestingly, in PDAC specimens, SIK1 was positively correlated with SLC7A11 expression, and PDAC counteracted ferroptosis through SIK1-mediated stabilization of HDAC5 and upregulation of SLC7A11 (Zhang et al., 2025). Thus, SLC7A11 is a key target in the regulation of ferroptosis, and the effect of reduced levels on cancer varies by type and microenvironment.

In conclusion, the current study validates that the silencing of FDFT1 can impede the ferroptosis via up-regulating SLC7A11 in MDA-MB-231 cells, potentially representing a target for therapeutic intervention against this malignancy. Furthermore, VPA’s ability to inhibit SLC7A11 levels via enhancing FDFT1 expression, facilitate ferroptosis in MDA-MB-231 cells, and curtail their proliferative capacity offers a novel direction for unraveling the mechanism of valproic acid in the treatment of TNBC. Meanwhile, our long-term objective entails conducting animal experiments to assess the impact of VPA on the induction of ferroptosis *in vivo* within TNBC cells, as well as its involvement in combating TNBC. Furthermore, we aim to investigate and refine tactics for the concurrent application of VPA and ferroptosis inducers within the context of animal research.

## Data Availability

The original contributions presented in the study are included in the article/supplementary material, further inquiries can be directed to the corresponding authors.
